# *Suhuang* Antitussive Capsule Ameliorates Corticosteroid Insensitivity in Cough Variant Asthma Guinea Pigs by Inhibiting p38 MAPK Signal Pathway

**DOI:** 10.1155/2022/1699429

**Published:** 2022-03-17

**Authors:** Tianyi Lyu, Demin Li, Haojun Zhang, Siyi Chen, Daowen Yang

**Affiliations:** ^1^Beijing University of Chinese Medicine, No. 11, Bei San Huan Dong Lu, Chaoyang District, Beijing 100029, China; ^2^Department of Traditional Chinese Medicine for Pulmonary Diseases, Center of Respiratory Medicine, China-Japan Friendship Hospital, Beijing, China; ^3^National Center for Respiratory Medicine, Beijing, China; ^4^Institute of Respiratory Medicine, Chinese Academy of Medical Sciences, Beijing, China; ^5^National Clinical Research Center for Respiratory Diseases, Beijing, China; ^6^WHO Collaborating Centre for Tobacco Cessation and Respiratory Diseases Prevention, No. 2, East Yinghua Road, Chaoyang District, Beijing 100029, China

## Abstract

**Methods:**

The CVA guinea pig model was successfully established by use of ovalbumin (OVA) sensitization and cigarette smoke (CS) exposure. The guinea pigs were divided into 6 groups: a control group, an OVA model group, an OVA + CS model group, a *Suhuang* treatment group, a BUD treatment group, and a combination (*Suhuang* and BUD) treatment group. The effects of the treatment were determined by measuring lung function (RI/Cydn) and cough symptoms (coughs number/cough latency) as outcome criteria. The levels of inflammatory cytokines in bronchoalveolar lavage fluid (BALF) were determined by ELISA. Lung tissues were stained by hematoxylin and eosin (H&E). The expressions of GR/total p38 MAPK/p-p38 MAPK were detected by Western blot. The MKP-1 mRNA levels were detected by RT-PCR.

**Results:**

Combination treatment significantly decreased RI/coughs numbers and increased Cydn/cough latency. Significantly, the results indicated that combination treatment decreased injury to pulmonary tissues. Results also revealed that levels of inflammatory cytokines were reduced in all treatment groups but most markedly in the combination treatment group. Moreover, *Suhuang* treatment significantly ameliorated corticosteroid insensitivity by improving the expression of glucocorticoid receptors (GR). The expressions of total p38 MAPK and p-p38 MAPK in lung tissue were significantly inhibited in the *Suhuang* and combination treatment groups. The MKP-1 mRNA levels in *Suhuang* and combination treatment groups were also increased significantly.

**Conclusion:**

*Suhuang* was effective for reversing corticosteroid insensitivity by regulating the p38 MAPK signal pathway, and combining BUD and *Suhuang* treatment showed synergistic interactions in CVA guinea pigs. Our findings showed that this combination therapy might be a promising therapeutic agent for CVA and also clarified its underlying mechanism of action, providing a theoretical basis for clinical combination treatment with *Suhuang* and BUD in CVA patients.

## 1. Introduction

Cough variant asthma (CVA) is a common medical condition that has significant effects on quality of life [1]. A prospective, multicenter survey demonstrated that nearly one-third of chronic cough cases were associated with CVA in China [2]. A Japanese case series showed that CVA was one of the primary causes of isolated chronic nonproductive cough [3]. Patients with CVA present chronic cough as their only typical symptom, which is associated with airway hyperresponsiveness (AHR) and airway inflammation [4]. Approximately 30%–40% of CVA cases will evolve into classic asthma [5]. Therefore, the early usage of inhaled corticosteroids (ICSs) is recommended as a treatment for CVA with persistent airway inflammation [6]. Unfortunately, although ICSs are the primary choices for asthma treatment, a certain proportion of patients remain symptomatic despite ICS treatment [5].

The commercially available product, *Suhuang* Antitussive Capsule (National Drug Standard for China, Z20103075), was created by Professor Chao Enxiang, a highly regarded doctor of traditional Chinese medicine (TCM). *Suhuang* Antitussive Capsule is composed of 9 traditional Chinese medicinals: *Ephedrae Herba* (Mahuang), *Perillae Folium* (Zisuye), *Pheretima* (Dilong), *Cicadae Periostracum* (Chantui), *Arctii Fructus* (Niubangzi), *Schisandrae Chinensis* Fructus (Wuweizi), *Peucedani Radix* (Qianhu), *Eriobotryae Folium* (Pipaye), and *Perillae Fructus* (Zisuzi) [7]. Previous studies demonstrated that this drug inhibited AHR and airway inflammation in chronic OVA-challenged asthma mice [8]. It was also proven to increase activity in the reticular endothelial system and to improve immune function in patients with CVA [9]. In addition, modern pharmacological experiments revealed that *Perillae Folium* (Zisuye), *Ephedrae Herba* (Mahuang), and *Cicadae Periostracum* (Chantui) had anti-inflammatory and antitussive effects [10]. *Ephedrae Herba* (Mahuang), one important component of *Suhuang*, has been shown to exert anti-inflammatory effects in various animal models or *in vitro* [11]. For example, San'ao Decoction has *Ephedrae Herba* as its main ingredient, and this was proven to inhibit asthmatic inflammation in mice through an upregulation of regulatory T cells and attenuation of IL-4-induced eotaxin expression in human airway epithelial cells [8, 11].

*Suhuang* was approved by the China Food and Drug Administration (CFDA) in 2008 and was recommended as the only Chinese Patent (prepared) Medicine to treat CVA in the “Chinese Thoracic Society 2015 Guidelines for the Diagnosis and Treatment of Cough” [12, 13]. *Suhuang* was recommended as a first-line treatment medication in the guidelines [13]. Recent evidence has also supported the hypothesis that certain single medicinal plants in *Suhuang*, particularly *Perillae Fructus* (Zisuzi) and *Pheretima* (Dilong), can alleviate airway inflammation through the p38 MAPK signal pathway [14, 15]. Moreover, several recent studies have indicated that the combination of ICS and *Suhuang* can effectively improve the efficacy rate in CVA patients compared with ICS or *Suhuang* monotherapy [16]. Despite the evidence for its significant clinical efficacy, the exact antitussive mechanisms of the combination treatment (*Suhuang* and BUD) still remain unclear.

Relative corticosteroid insensitivity is one of the most common reasons why patients respond poorly to ICS in clinical practice [17]. In separate research, p38 MAPK has recently been identified as a critical kinase for ameliorating corticosteroid insensitivity [18]. The level of p38 MAPK phosphorylation was markedly upregulated in corticosteroid-insensitive patients [19]. Therefore p38 MAPK inhibitors have been suggested as potential treatments for asthma due to their role in downregulating p38 MAPK phosphorylation [20]. For example, MKP-1, a p38 MAPK-related phosphatase, is considered as an endogenous p38 MAPK inhibitor and appears to ameliorate the effects of glucocorticoids [21].

Therefore, this study aims to determine whether *Suhuang* could enhance the effects of BUD on corticosteroid-insensitive CVA guinea pigs by ameliorating corticosteroid insensitivity via the p38 MAPK signal pathway.

## 2. Materials and Methods

### 2.1. Reagents and Materials

Guinea pig IL-8 ELISA Kit (cat. number CBS-E13096Gp) and guinea pig TNF-*α* ELISA Kit (cat. number CBS-E06772Gu) were purchased from CUSABIO. Antiglucocorticoid Receptor [GR] antibody (cat. number ab2768) was purchased from Abcam. The p38 MAPK antibody (cat. number #9212) was purchased from CSL. Phospho-p38 MAPK antibody (cat. number YP0338) was purchased from ImmunoWay.

### 2.2. Preparation of Suhuang

*Suhuang* was purchased from Yangtze River Pharmaceutical Group (Beijing, China). It is comprised of 9 traditional Chinese medicinals. Based on previously published research [8], *Suhuang* was converted to an equivalent dose for adult guinea pigs at 3 g/kg and prepared as a 0.5 g/100 ml solution with NS before being administered to the animals.

### 2.3. Animals and Care

A total of 48 male and female guinea pigs, 5–7 weeks old, weighing 280–320 g, were purchased from Beijing Keyu Animal Breeding Center [SCXK (Beijing) 2018-0010]. The guinea pigs were maintained in a specific pathogen-free (SPF) environment with a temperature of 20–26°C, daily temperature fluctuation ≤4°C, humidity of 40–70%, 12 h light/dark cycle, and free access to food and water. All animal procedures performed in this study were approved by the Institute of Chinese Materia Medica, China Academy of Chinese Medical Sciences [SYXK (Beijing) 2019-0002].

### 2.4. Animal Groups and Model

All guinea pigs were randomly divided into 6 groups as follows (*n* = 8): a control group, an OVA model group, an OVA + CS model group, a *Suhuang* (3 g/kg) treatment group, a BUD treatment group, and a combination (*Suhuang* and BUD) treatment group.

**Figure 1:**
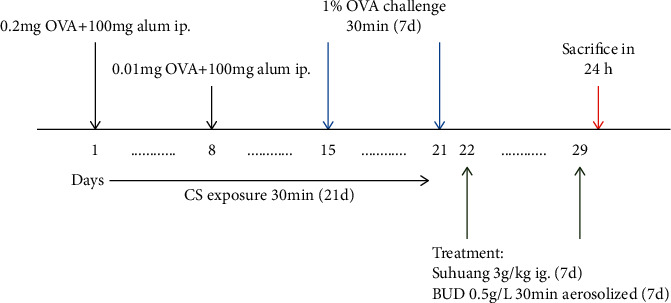
Timeline representation of the CVA guinea pig model and pharmacological interventions.

Guinea pigs in the OVA model group and OVA + CS model group were sensitized on day 1 (0.2% OVA-NS) and day 8 (0.01% OVA-NS) by intraperitoneal (ip.) injection of OVA in 0.1 ml and an equal volume of aluminum hydroxide. 7 days after the second sensitization, the guinea pigs were challenged with aerosolized 1% OVA-NS for 30 min per day over a week. The control group was administered NS instead. Guinea pigs from OVA + CS model group were exposed to CS for 0.5 h every day on day 1 to 21 at the same time. One hour after the last challenge, *Suhuang* treatment group and combination treatment group were given *Suhuang* treatment (3 g/kg) through intragastric (ig.) administration for 7 days. The BUD treatment group and combination treatment group were given aerosolized 8 ml BUD (1 mg/2 ml) treatment for 7 days at the same time. Sensitization, challenge, and treatment protocols for the different groups in this study are summarized in Figure 1.

### 2.5. Sample Collection

The guinea pigs were anesthetized by intraperitoneal injection of 5% pentobarbital (0.3 mL/100 g) 24 hours after the last drug administration. BALF was collected immediately using PBS lavaged to the right lung 3 times by gentle cannulation. The upper lobes of the left lungs were fixed in 10% neutral formalin and the inferior lobes of the left lungs were collected and frozen at –80°C.

### 2.6. Cough Measurement

Each guinea pig was placed in a transparent sealed chamber and exposed to capsaicin aerosol for cough challenges 60 min after the last drug administration for its respective group. A mixed airflow of capsaicin aerosol (10^−4^ mol/L) was blown into the chamber through a tube that bought it in one end and out the opposite end. Five minutes later, the mixed airflow of capsaicin aerosol in the chamber was neutralized with fresh air. The number of coughs over 10 min was recorded. In addition, the time from the start of the aerosol inhalation to the first cough was measured as the cough latency. The cough could be recognized from the characteristic posture (splaying of the front feet and forward stretching of the neck and opening of the mouth) and the cough sounds were also amplified by a wireless microphone placed in the box. The number of coughs and cough latency were recorded and analyzed by two trained observers.

### 2.7. Assessment of Lung Function

Curative effects of drug treatment on lung function were estimated with the RC system (Buxco Electronics, NC, USA). Guinea pigs were anesthetized with 3% pentobarbital sodium and intubated so that they could be placed in a whole body plethysmography chamber and then exposed to increasing concentrations of methacholine (2, 4, 6, and 8 mg/ml). Data on airway resistance (RI) and lung compliance (Cydn) were collected and assessed based on the RC system.

### 2.8. Enzyme-Linked Immunosorbent Assay (ELISA)

The levels of interleukin-8 (IL-8) and Tumor Necrosis Factor-*α* (TNF-*α*) in the BALF supernatant were determined by using enzyme-linked immunosorbent assay (ELISA) following the manufacturer's instructions (CUSABIO, Beijing, China).

### 2.9. Histologic Evaluation

The pulmonary tissues were sectioned (4 *μ*m), dewaxed, and dehydrated in decreasing concentrations of ethanol. They were then rinsed with distilled water and stained using hematoxylin and eosin (H&E). Microscopic images of stained sections were obtained using a microscope (Olympus, Japan) at ×100 magnification.

### 2.10. The Score Criterion of Ashcroft Grading Scale

The H&E stained histological sections were analyzed by visual assessment via Ashcroft grading scale [19]. The features of the Ashcroft grading scale are shown in Table 1.

### 2.11. Western Blot

After the guinea pigs were sacrificed, the frozen and purified lung tissues were prepared, which were homogenized in lysis buffer. Then, the supernatant was collected and centrifuged at 12,000 rpm for 5 min at 4°C. Before preparing protein samples, the concentration of each sample was measured by using the BCA protein assay. Equal amounts of the extracted proteins (20 *μ*g) in lysis buffer were separated by SDS-PAGE gel and transferred onto a PVDF membrane. Subsequently, the membranes were treated with the indicated primary antibodies overnight at 4°C and then incubated with the HRP-coupled secondary antibody. Western blotting analysis for Antiglucocorticoid Receptor (Abcam 1 : 200), anti-p38 MAPK (CST 1 : 2000), and anti-pp38 MAPK (ImmunoWay 1 : 1,000) antibody was used to measure specific antibodies. Relative expression levels of each protein were normalized to endogenous control *β*-tubulin using ImageJ software.

### 2.12. Quantitative Real-Time PCR

Total RNA samples were taken from the lungs after stimulation, using the HiPure Total RNA Mini Kit (Magen China) according to manufacturer's protocol for extraction. 1*μ*g RNA was reverse-transcribed to cDNA in 20 *μ*l using A3500 Reverse Transcription System (Promega, United States). Quantitative real-time PCR was performed using SYBR^®^ Green Real-Time PCR Master Mix. The primer of MKP-1 and *β*-actin was designed by GeneCopoeia, Inc.

### 2.13. Statistical Analysis

All graphing and statistical analyses were performed using GraphPad Prism 8.0 (GraphPad Software, San Diego, CA, USA). If the data showed normal distribution, the results were shown as mean ± SEM and scatter plot. Otherwise, the results were shown as median (interquartile range, P75–P25) and scatter plot. Comparisons among multiple groups were analyzed with one-way ANOVA. Single comparisons were made with the Sidak test. If the data did not meet normal distribution, comparisons among multiple groups were analyzed with nonparametric analysis. Single comparisons were made with the Dunn test. Statistical significance was considered at *P* < 0.05 (NS, not significant; ^*∗*^*P* < 0.05, ^*∗∗*^*P* < 0.01, and ^*∗∗∗*^*P* < 0.001).

## 3. Results

### 3.1. Combination Treatment Improved Cough Symptoms in the CVA Guinea Pig Model

**Figure 2:**
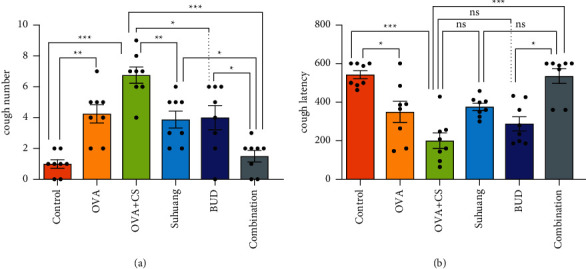
Cough symptoms regulated by treatment. (a) Coughs number of guinea pigs. (b) Cough latency of guinea pigs. Normal distribution, data are presented as mean ± SEM and scatter plot (*n* = 8, no values were excluded). OVA: ovalbumin, OVA + CS: ovalbumin + cigarette smoke exposure, *Suhuang*: *Suhuang* treatment, BUD: BUD treatment, Combination: *Suhuang* + BUD treatment. ns, *P* < 0.05, ^*∗*^*P* < 0.05, ^*∗∗*^*P* < 0.01, and ^*∗∗∗*^*P* < 0.001.

We observed the effects of *Suhuang* on cough symptoms (Figure 2). Compared with the control group, the OVA + CS model group exhibited significantly increased number of coughs and decreased cough latency (*P* < 0.001). Results also revealed that the number of coughs in the treatment groups was significantly reduced and the cough latency in the combination treatment group increased (*P* < 0.001). However, there was no significant difference between the *Suhuang* or BUD treatment group and the OVA + CS model group in terms of cough latency (*P* > 0.05).

### 3.2. Combination Treatment Prevented AHR and Increased Cydn in the CVA Guinea Pig Model

**Figure 3:**
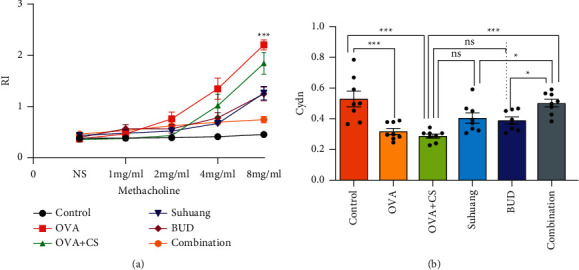
Lung function regulated by treatment. (a) RI of guinea pigs. (b) Cydn of guinea pigs. Data are presented as mean ± SEM and scatter plot (*n* = 8, no values were excluded). OVA: ovalbumin, OVA + CS: ovalbumin + cigarette smoke exposure, *Suhuang*: *Suhuang* treatment, BUD: BUD treatment, Combination: *Suhuang* + BUD treatment. ns, *P* < 0.05, ^*∗*^*P* < 0.05, ^*∗∗*^*P* < 0.01, and ^*∗∗∗*^*P* < 0.001.

We observed the effects of combination treatment on RI and Cydn (Figure 3). Airway hyperresponsiveness (AHR) is considered to be the major symptom of CVA, so documentation of variability in lung function is important [20]. An invasive method was undertaken to determine the airway resistance (RI) and lung compliance (Cydn). Compared with the control group, RI significantly increased and Cydn significantly decreased in the OVA + CS model group. Treatments (*Suhuang*, BUD, and combination) significantly decreased RI at a dose of 8 mg/ml Mch (NS versus OVA, *P* < 0.001; NS versus CS + OVA, *P* < 0.001; CS + OVA versus *Suhuang*, *P* < 0.05; CS + OVA versus BUD, *P* < 0.05; CS + OVA versus combination, *P* < 0.001; *Suhuang* versus combination, *P* < 0.05; BUD versus combination, *P* < 0.05). Moreover, combination treatments significantly increased Cydn compared with the OVA + CS model group (*P* < 0.001).

### 3.3. Combination Treatment Decreased the Expression of Inflammatory Cytokines in the CVA Guinea Pig Model

**Figure 4:**
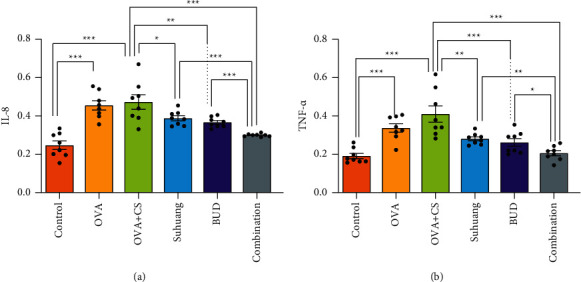
Inflammatory cytokines regulated by treatment. (a) IL-8 of guinea pigs (b) TNF-*α* of guinea pigs. Data are presented as mean ± SEM and scatter plot (*n* = 8, no values were excluded). OVA: ovalbumin, OVA + CS: ovalbumin + cigarette smoke exposure, *Suhuang*: *Suhuang* treatment, BUD: BUD treatment, Combination: *Suhuang* + BUD treatment. ns, *P* < 0.05, ^*∗*^*P* < 0.05, ^*∗∗*^*P* < 0.01, and ^*∗∗∗*^*P* < 0.001.

The levels of IL-8 and TNF-*α* were determined using ELISA (Figure 4). Compared with the control group, the expressions of IL-8 and TNF-*α* were increased in the OVA + CS model group (*P* < 0.01). Compared with the OVA + CS model group, combination treatment significantly decreased the secretion of IL-8 and TNF-*α* (*P* < 0.001).

### 3.4. Combination Treatment Alleviated Pulmonary Tissues Injury in the CVA Guinea Pig Model

**Figure 5:**
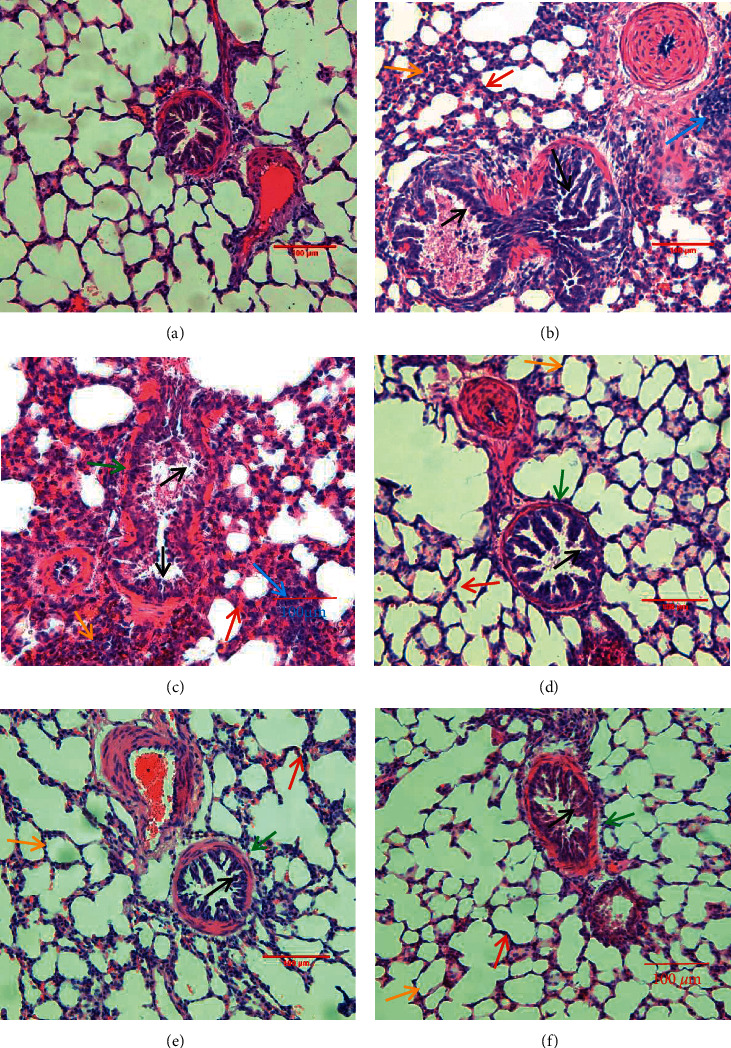
Pathological changes of lung tissues. H&E staining slices from lung sections, magnification 100×. (a) Control group, (b) OVA group, (c) OVA + CS group, (d) *Suhuang* treatment group, (e) BUD treatment group, and (f) combination treatment group. In Figures 5(b) and 5(c), we observe widening of alveolar septum (red arrows), destruction of alveolar structure (yellow arrows), dilation of tracheal lumen (green arrows), proliferation or flatness of mucosal folds (black arrows), and aggregation of inflammatory cells (blue arrows). In Figures 5(d)–5(f), we observe widening of alveolar septum (red arrows), destruction of alveolar structure (yellow arrows), dilation of tracheal lumen (green arrows), proliferation or flatness of mucosal folds (black arrows), and aggregation of inflammatory cells are reduced.

**Figure 6:**
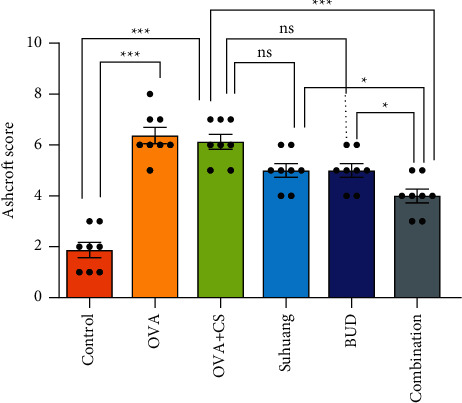
Ashcroft score regulated by treatment. Data are presented as mean ± SEM and scatter plot (*n* = 8, no values were excluded). OVA: ovalbumin, OVA + CS: ovalbumin + cigarette smoke exposure, *Suhuang*: *Suhuang* treatment, BUD: BUD treatment, Combination: Suhuang + BUD treatment. ns, *P* < 0.05, ^*∗*^*P* < 0.05, ^*∗∗*^*P* < 0.01, and ^*∗∗∗*^*P* < 0.001.

Pulmonary pathology was examined by staining with HE (Figure 5). The Ashcroft scale characterizations were used to analyze H&E stained histological sections (Figure 6 and Table 1) [22]. Infiltration of inflammatory cells, changes in alveolar structure, dilation of trachea, and pathological changes of mucosal folds are common pathophysiological features of pulmonary tissues in cough variant asthma [23]. In the lung tissues of the OVA + CS group, aggregation of inflammatory cells, destruction of alveolar structure, dilation of tracheal lumen, widening of alveolar septum, and changes of mucosal folds were observed. Treatments reduced the infiltration of inflammatory, destruction of alveolar structure, dilation of the trachea, and pathological changes of mucosal folds. Further analysis of pathological changes in the lung tissues revealed that the scores on the Ashcroft grading scale were significantly reduced in combination treatment group, compared with the OVA + CS model group (*P* < 0.001).

### 3.5. Suhuang Increased Corticosteroid Insensitivity by Upregulating the Expression of Glucocorticoid Receptors

**Figure 7:**
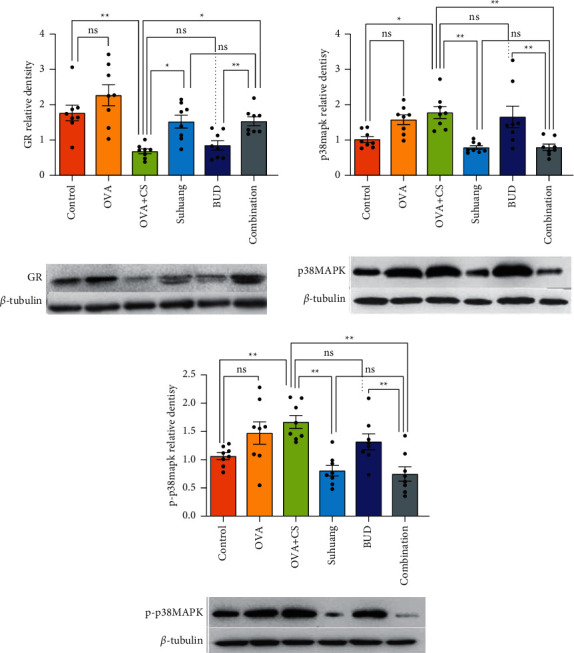
The levels of GR, p38 MAPK, and p-p38 MAPK proteins in lung tissues. Data are presented as mean ± SEM and scatter plot (*n* = 8, no values were excluded). OVA: ovalbumin, OVA + CS: ovalbumin + cigarette smoke exposure, *Suhuang*: *Suhuang* treatment, BUD: BUD treatment, Combination: *Suhuang* + BUD treatment. ns, *P* < 0.05, ^*∗*^*P* < 0.05, ^*∗∗*^*P* < 0.01, and ^*∗∗∗*^*P* < 0.001.

Many studies have indicated that cigarette smoke can induce corticosteroid insensitivity in asthmatic airways [24]. We established a CVA corticosteroid insensitivity guinea pig model through cigarette smoke exposure and OVA-sensitization. As is well known, glucocorticoids (GCs) act by binding to a cytosolic GR, so GR is a key protein associated with corticosteroid resistance [25]. In this study, we analyzed the expressions of glucocorticoid receptor (GR) by western blot (Figure 7). Compared with control group, the expressions of GR were slightly increased in the OVA group. Importantly, compared with the OVA model group, the expressions of GR were significantly decreased in the CS + OVA model group.

Our study investigated whether *Suhuang* could reverse corticosteroid insensitivity by measuring the expressions of GR. Results revealed that *Suhuang* treatment significantly increased the expressions of GR (NS versus OVA, *P* < 0.05; OVA versus CS + OVA, *P* < 0.05; CS + OVA versus *Suhuang*, *P* < 0.05; CS + OVA versus BUD, *P* < 0.05; CS + OVA versus combination, *P* < 0.05).

### 3.6. Suhuang Upregulated the Expression of Glucocorticoid Receptor by p38 MAPK Signaling Pathway

Increased p38 MAPK activation is a feature of epithelial cells from patients with asthma [26]. Moreover, the p38 MAPK activation has been proven to alter corticosteroid responsiveness. The levels of activation of p38 MAPK were calculated by the expressions of phospho-p38 MAPK (p-p38 MAPK) and total p38 MAPK [27]. The expressions of p-p38 MAPK and total p38 MAPK were measured by western blot (Figure 7). Compared with the control group, the expressions of p-p38 MAPK and total p38 MAPK were increased in the OVA + CS model group. Results also revealed that *Suhuang* treatment significantly decreased the expressions of p-p38 MAPK and total p38 MAPK (NS versus OVA, *P* < 0.05; OVA versus CS + OVA, *P* < 0.05; CS + OVA versus *Suhuang*, *P* < 0.01; CS + OVA versus Budesonide, *P* < 0.05; CS + OVA versus combination, *P* < 0.01).

**Figure 8:**
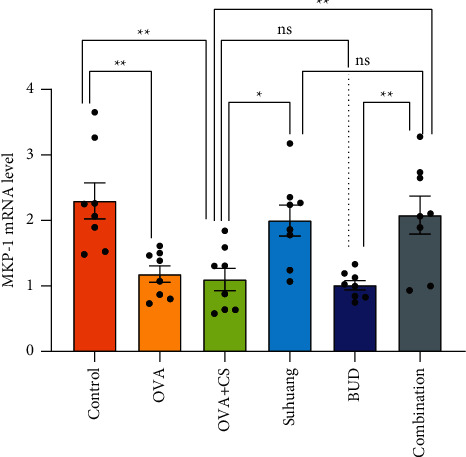
The levels of MKP-1 mRNA in lung tissues. Data are presented as mean ± SEM and scatter plot (*n* = 8, no values were excluded). OVA: ovalbumin, OVA + CS: ovalbumin + cigarette smoke exposure, *Suhuang*: *Suhuang* treatment, BUD: BUD treatment, Combination: *Suhuang* + BUD treatment. ns, *P* < 0.05, ^*∗*^*P* < 0.05, ^*∗∗*^*P* < 0.01, and ^*∗∗∗*^*P* < 0.001.

MKP-1, also called DUSP, is reported to dephosphorylate p38 MAPK, thereby suppressing the downstream signaling of these kinases [21]. We therefore hypothesized that MKP-1 was involved in p38 MARK suppression by *Suhuang.* The current research tested this hypothesis by measuring the mRNA levels of MKP-1 by qRT-PCR (Figure 8). Compared with the CS + OVA model group, the *Suhuang* treatment significantly decreased the mRNA levels of MKP-1 (NS versus OVA, *P* < 0.01; OVA versus CS + OVA, *P* < 0.01; CS + OVA versus *Suhuang*, *P* < 0.05; CS + OVA versus BUD, *P* < 0.05; CS + OVA versus combination, *P* < 0.01).

## 4. Discussion

CVA is a chronic inflammatory disease. Persistent inflammation has been implicated to cause structural changes and remodeling of the airway, characterized by histopathological changes such as infiltration of inflammatory cells, destruction in alveolar structure, dilation of the trachea, and pathological changes of mucosal folds [28]. These histopathological changes commonly lead to pulmonary dysfunction, such as airway hyperresponsiveness (AHR) and chronic cough [29].

Cytokines IL-8 and TNF-*α* were studied in this experiment as inflammatory markers. There is experimental evidence of both IL-8 and TNF-*α* being produced by airway epithelial cells following inflammatory stimuli in asthmatic patients. In addition, the production of IL-8 and TNF-*α* is regulated by p38 MAPK signal pathway [21, 30].

**Figure 9:**
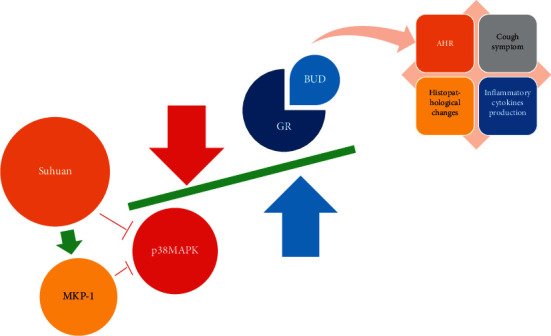
The graphic illustration of the antitussive mechanism of *Suhuang*. *Suhuang* increased expression of GR in a manner dependent on inhibition of p38 MAPK phosphorylation and activity by increasing MKP-1 level. Therefore, combination treatment of *Suhuang* and BUD showed better improvement in AHR, cough symptom, histopathological changes, and inflammatory cytokines production.

*Suhuang* has been used for long-term treatment of CVA in China for a number of years [31]. The BUD regime is also commonly administered for the treatment of CVA [32]. Our study investigated whether combination treatment of *Suhuang* and BUD could show synergistic interaction in CVA guinea pigs (Figure 9).

The purpose of this study is to examine whether combination treatment could prevent AHR and ameliorate cough symptoms. Our results showed that combination treatment prevented AHR via decreasing RI and increasing Cdyn and ameliorated cough symptom via reducing the number of coughs and increasing the cough latency. Meanwhile, combination treatment inhibited lung histopathological changes such as infiltration of inflammatory cells, destruction in alveolar structure, dilation of the trachea, and pathological changes of mucosal folds. Our results also showed a significant difference in the reduction in the levels of TNF-*α* and IL-8 mediated by combination treatment. Importantly, the combination treatment showed better improvement in AHR, cough symptom, histopathological changes, and inflammatory cytokines production compared to monotherapy of BUD or *Suhuang*. These results clearly showed that combining *Suhuang* and BUD created synergistic interactions in CVA guinea pigs.

Corticosteroid insensitivity is one of the most common reasons why patients respond poorly to ICS [17]. We hypothesized that one of the effects of *Suhuang* was that it could improve corticosteroid insensitivity, so combining *Suhuang* and BUD would show demonstrable synergistic interactions in CVA.

The OVA-sensitization CVA model was well accepted and widely reported in previous studies [33]. Here, our study generated guinea pig models of CVA with ovalbumin (OVA) as the allergen. Otherwise, the existing results show that cigarette smoke is an important cause of corticosteroid insensitivity [24]. Neutrophilic airway inflammation, decreased expression of GR-*α*, and activity of histone deacetylase 2 (HDAC2) are presumed to be involved in smoking-related corticosteroid insensitivity [34]. Hence, the guinea pig CVA model of corticosteroid insensitivity was established by OVA-sensitization and CS exposure. In light of our observation of corticosteroid insensitivity, these results reported that GR expression in OVA + CS guinea pig model was significantly reduced compared with that in control group. Thus, a guinea pig CVA model of corticosteroid insensitivity was successfully established.

The anti-inflammatory effects of ICS are mediated through the GR, a ligand-activated transcription factor that modulates both inflammatory and anti-inflammatory gene expressions. After ICS binds to GR, GR dissociates from chaperone proteins and rapidly translocates into the nucleus, where GR either binds to specific glucocorticoid-responsive elements (GRE) or represses transcription of proinflammatory genes by interacting with inflammatory transcription factors [35, 36]. Our research demonstrated that *Suhuang* effectively upregulated the expression of GR in CVA guinea pig model. This raised the possibility that *Suhuang* improved corticosteroid responsiveness.

The mitogen-activated protein kinase (MAPK) family is serine/threonine family of kinases, and p38 MAPK is one of the most important members of MAPK family [37]. GR expression can be mediated by p38 MAPK, indicative of critical effect of p38 MAPK in mediating steroid resistance. Recent studies reported heightened p38 MAPK phosphorylation and activity in lung cells [26, 38]. In addition, inhibition of p38 MAPK phosphorylation and activity led to reversal of corticosteroid insensitivity. To investigate the effect of *Suhuang* on p38 MAPK, the expressions of total p38 MAPK and p-p38 MAPK were measured by western blot. These data confirmed that *Suhuang* decreased the expressions of total p38 MAPK and p-p38 MAPK, suggesting that *Suhuang* improved corticosteroid responsiveness by decreasing total p38 MAPK and p-p38 MAPK expressions. MKP-1 is reported to dephosphorylate p38 MAPK, thereby suppressing the downstream signaling of these kinases [21]. In our study, the mRNA levels of MKP-1 in the *Suhuang* and combination treatment groups showed significant improvement compared with the OVA + CS group. It thus provides new insights, suggesting that *Suhuang* mediated p38 MAPK signal pathway via decreasing MKP-1 levels, and thus it may help in the development of new therapeutic strategies for the improvement of corticosteroid responsiveness.

## 5. Conclusion

In summary, our study demonstrated that the combination treatment of BUD and *Suhuang* can effectively decrease AHR, ameliorate cough symptoms, alleviate pulmonary tissue injury, and improve airway inflammation. Furthermore, *Suhuang* had the effect of reversing corticosteroid insensitivity by regulating p38 MAPK signaling pathways; therefore combining BUD and *Suhuang* showed synergistic interaction in CVA. In addition, observations suggest that *Suhuang* mediated p38 MAPK signal pathways via increasing MKP-1 levels. The results of this study demonstrate that combination treatment with *Suhuang* and BUD is a promising new candidate for CVA therapy, and we clarified its underlying mechanism. Moreover, our findings support a possible application of *Suhuang* as corticosteroid insensitivity reverser and p38 MAPK inhibitor in clinical settings.

## Figures and Tables

**Table 1:** Ashcroft score.

Grade of fibrosis	Manifestations
0	Alveolar septa: no fibrotic burden at the most flimsy small fibres in some alveolar walls
	Lung structure: normal lung
1	Alveolar septa: isolated gentle fibrotic changes (septum <3× thicker than normal)
	Lung structure: alveoli partly enlarged and rarefield, but no fibrotic masses present
2	Alveolar septa: clearly fibrotic changes (septum >3× thicker than normal) with knot like formation but not connected to each other
	Lung structure: alveoli partly enlarged and rarefield, but no fibrotic masses present
3	Alveolar septa: contiguous fibrotic walls (septum >3× thicker than normal) predominantly in whole microscopic field
	Lung structure: alveoli partly enlarged and rarefield, but no fibrotic masses present
4	Alveolar septa: variable
	Lung structure: single fibrotic masses (<10% microscopic field)
5	Alveolar septa: variable
	Lung structure: single fibrotic masses (>10% and <50% microscopic field). Lung structure severely damaged but still preserved
6	Alveolar septa: variable, mostly not existent
	Lung structure: large contiguous fibrotic masses (>50% microscopic field). Lung structure mostly not preserved
7	Alveolar septa: nonexistent
	Lung structure: alveoli nearly obliterated with fibrous masses but still up to five air bubbles
8	Alveolar septa: nonexistent
	Lung structure: microscopic field with complete obliteration with fibrotic masses

## Data Availability

The data used to support the findings of this study are available from the first author upon request.

## Supplementary Materials

Supplementary Materials Supplementary Table S1: three-line table for cough symptoms of guinea pigs in six groups. Supplementary Table S2: three-line table for RI of guinea pigs in six groups. Supplementary Table S3: three-line table for Cydn of guinea pigs in six groups. Supplementary Table S4: three-line table for inflammatory cytokines of guinea pigs in six groups. Supplementary Table S5: three-line table for Ashcroft score of guinea pigs in six groups. Supplementary Table S6: three-line table for GR, total p38 MAPK, and p-p38 MAPK levels of guinea pigs in six groups. Supplementary Table S7: three-line table for mRNA MKP-1 level of guinea pigs in six groups. Supplementary Figure S1: levels of GR proteins in lung tissues. Supplementary Figure S2: levels of total p38 MAPK proteins in lung tissues. Supplementary Figure S3: levels of p-p38 MAPK proteins in lung tissues. Supplementary Figure S4: aggregation of inflammatory cells (red circle) in H&E staining slices from lung sections: (a) OVA group; (b) OVA + CS group.
