# Supplementary material for: Suhuang Antitussive Capsule Ameliorates Corticosteroid Insensitivity in Cough Variant Asthma Guinea Pigs by Inhibiting p38 MAPK Signal Pathway
Source: Evid Based Complement Alternat Med. 2022 Mar 17;2022:1699429. doi: 10.1155/2022/1699429 (PMC8947934; doi:10.1155/2022/1699429)

Table 1 Cough symptoms of guinea pigs in six groups

| **Groups** | **Cough number in 10 minutes（freq）** | **Cough latency（s）** |
| --- | --- | --- |
| **Control group** | **1.00±0.27** | **544.00(110.00)** |
| **OVA model group** | **4.25±0.59^**^** | **375.50(275.30)** |
| **OVA+CS model group** | **6.75±0.53^***^** | **184.00(147.50)^***^** |
| **Suhuang treatment group** | **3.88±0.55^*^^** | **379.50(78.70)** |
| **BUD treatment group** | **4.00±0.78^**^^** | **257.50(220.50)^^^** |
| **Combination treatment group** | **1.50±0.38^∆∆∆^** | **595.00(188.50)^∆∆∆^** |

Note. Compared with control group, *P<0.05, **P<0.01，***P<0.001. Compared with OVA+CS model group, ^∆^P<0.05, ^∆∆^P<0.01，^∆∆∆^P<0.001. Compared with combination treatment group, ^P<0.05, ^^P<0.01，^^^P<0.001

Table 2 RI of guinea pigs in six groups

| **Groups** | **NS** | **0.2mg/mL** | **0.4mg/mL** | **0.6mg/mL** | **0.8mg/mL** |
| --- | --- | --- | --- | --- | --- |
| **Control group** | **0.38±0.03** | **0.39±0.03** | **0.39±0.03** | **0.41±0.02** | **0.46±0.02** |
| **OVA model group** | **0.37±0.03** | **0.47±0.03** | **0.76±0.13^**^** | **1.35±0.21^***^** | **2.21±0.10^***^** |
| **OVA+CS model group** | **0.35±0.03** | **0.38±0.03** | **0.44±0.03** | **1.02±0.22^*^** | **1.85±0.21^***^** |
| **Suhuang treatment group** | **0.44±0.03** | **0.48±0.04** | **0.52±0.04** | **0.68±0.05** | **1.22±0.15^∆^^** |
| **BUD treatment group** | **0.40±0.04** | **0.57±0.08** | **0.57±0.08** | **0.78±0.10** | **1.25±0.13^∆^^** |
| **Combination treatment group** | **0.47±0.04** | **0.53±0.04** | **0.63±0.03** | **0.70±0.06** | **0.79±0.06^∆∆∆^** |

Note. Compared with control group, *P<0.05, **P<0.01，***P<0.001. Compared with OVA+CS model group, ^∆^P<0.05, ^∆∆^P<0.01，^∆∆∆^P<0.001. Compared with combination treatment group, ^P<0.05, ^^P<0.01，^^^P<0.001

Table 3 Cydn of guinea pigs in six groups

| **Groups** | **Cydn** |
| --- | --- |
| **Control group** | **0.53±0.05** |
| **OVA model group** | **0.32±0.02^***^** |
| **OVA+CS model group** | **0.29±0.01^***^** |
| **Suhuang treatment group** | **0.41±0.03^^^** |
| **BUD treatment group** | **0.39±0.02^^^** |
| **Combination treatment group** | **0.50±0.03^∆∆^** |

Note. Compared with control group, *P<0.05, **P<0.01，***P<0.001. Compared with OVA+CS model group, ^∆^P<0.05, ^∆∆^P<0.01，^∆∆∆^P<0.001. Compared with combination treatment group, ^P<0.05, ^^P<0.01，^^^P<0.001

Table 4 Inflammatory cytokines of guinea pigs in six groups

| **Groups** | **IL-8** | **TNF-α** |
| --- | --- | --- |
| **Control group** | **0.25±0.02** | **0.20±0.01** |
| **OVA model group** | **0.46±0.02^***^** | **0.34±0.02^***^** |
| **OVA+CS model group** | **0.47±0.04^***^** | **0.41±0.04^***^** |
| **Suhuang treatment group** | **0.39±0.01^∆^^^^** | **0.28±0.01^∆∆^^^** |
| **BUD treatment group** | **0.37±0.01^∆∆^^^^** | **0.26±0.02^∆∆∆^^** |
| **Combination treatment group** | **0.30±0.00^∆∆∆^** | **0.20±0.01^∆∆∆^** |

Note. Compared with control group, *P<0.05, **P<0.01，***P<0.001. Compared with OVA+CS model group, ^∆^P<0.05, ^∆∆^P<0.01，^∆∆∆^P<0.001. Compared with combination treatment group, ^P<0.05, ^^P<0.01，^^^P<0.001

Table 5 Ashcroft Score of guinea pigs in six groups

| **Groups** | **Ashcroft Score** |
| --- | --- |
| **Control group** | **1.86±0.30** |
| **OVA model group** | **6.38±0.32^***^** |
| **OVA+CS model group** | **6.13±0.30^***^** |
| **Suhuang treatment group** | **5.00±0.27^^^** |
| **BUD treatment group** | **5.00±0.27^^^** |
| **Combination treatment group** | **4.00±0.27^∆∆∆^** |

Note. Compared with control group, *P<0.05, **P<0.01，***P<0.001. Compared with OVA+CS model group, ^∆^P<0.05, ^∆∆^P<0.01，^∆∆∆^P<0.001. Compared with combination treatment group, ^P<0.05, ^^P<0.01，^^^P<0.001

Table 6 Proteins GR,total p38MAPK,p-p38MAPK of guinea pigs in six groups

| **Groups** | **GR** | **total p38MAPK** | **p-p38MAPK** |
| --- | --- | --- | --- |
| **Control group** | **1.77±0.22** | **1.02±0.08** | **1.06±0.06** |
| **OVA model group** | **2.27±0.30** | **1.58±0.14** | **1.47±0.20** |
| **OVA+CS model group** | **0.68±0.07^**^** | **1.78±0.17^*^** | **1.67±0.12^*^** |
| **Suhuang treatment group** | **1.52±0.18^∆^** | **0.79±0.05^∆∆^** | **0.81±0.10^∆∆∆^** |
| **BUD treatment group** | **0.85±0.13^^^^** | **1.66±0.30^^^^** | **1.32±0.14^^^^** |
| **Combination treatment group** | **1.53±0.13^∆^** | **0.80±0.09^∆∆^** | **0.75±0.13^∆∆∆^** |

Note. Compared with control group, *P<0.05, **P<0.01，***P<0.001. Compared with OVA+CS model group, ^∆^P<0.05, ^∆∆^P<0.01，^∆∆∆^P<0.001. Compared with combination treatment group, ^P<0.05, ^^P<0.01，^^^P<0.001

Table 7 mRNA MKP-1 level of guinea pigs in six groups

| **Groups** | **MKP-1** |
| --- | --- |
| **Control group** | **2.30±0.28** |
| **OVA model group** | **1.18±0.12^**^** |
| **OVA+CS model group** | **1.10±0.17^**^** |
| **Suhuang treatment group** | **2.00±0.24^∆^** |
| **BUD treatment group** | **1.01±0.07^^^^** |
| **Combination treatment group** | **2.10±0.30^∆∆^** |

Note. Compared with control group, *P<0.05, **P<0.01，***P<0.001. Compared with OVA+CS model group, ^∆^P<0.05, ^∆∆^P<0.01，^∆∆∆^P<0.001. Compared with combination treatment group, ^P<0.05, ^^P<0.01，^^^P<0.001

Figure1 levels of GR proteins in lung tissues


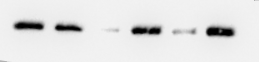

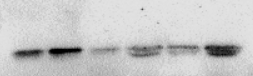

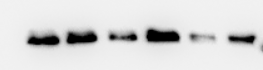


Figure2 levels of total p38MAPK proteins in lung tissues


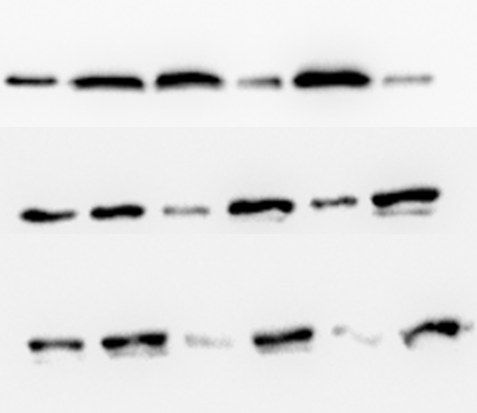


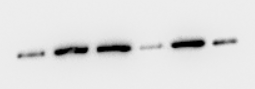
Figure3 levels of p-p38MAPK proteins in lung tissues


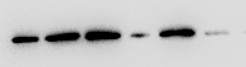

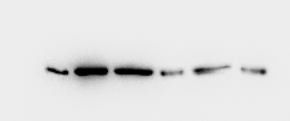
Figure 4 Aggregation of inflammatory cells(red circle) in ova group and ova+cs group HE staining slices from lung sections


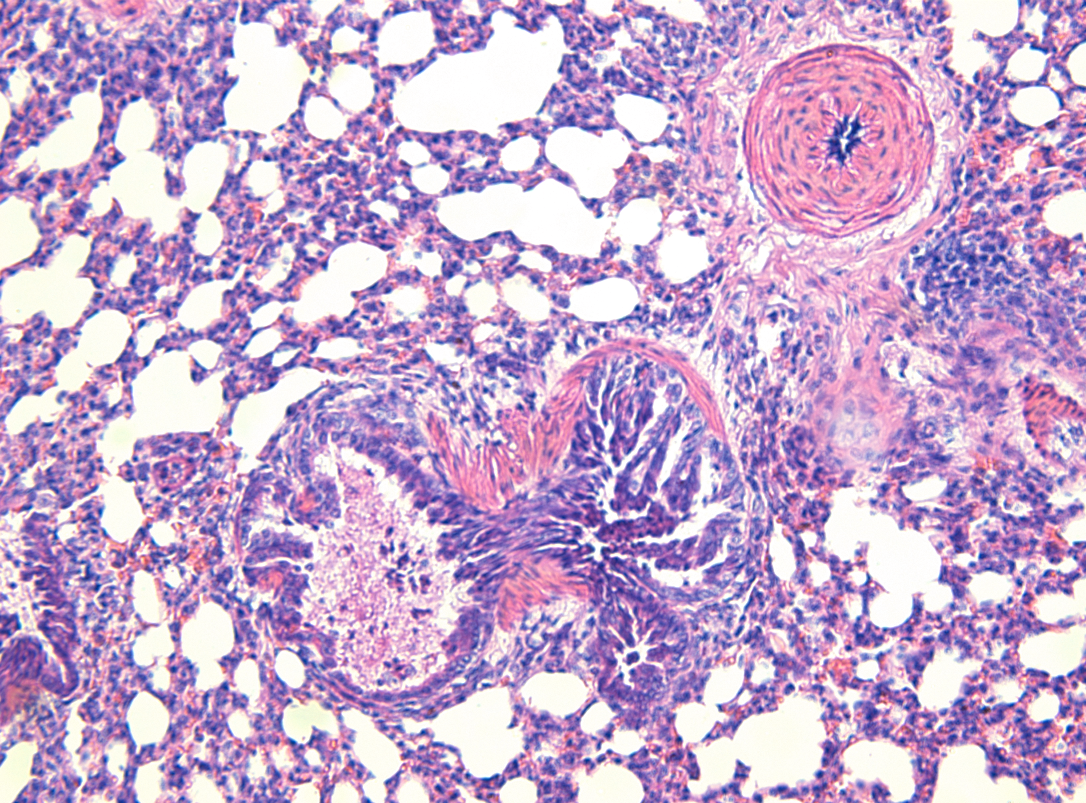


B


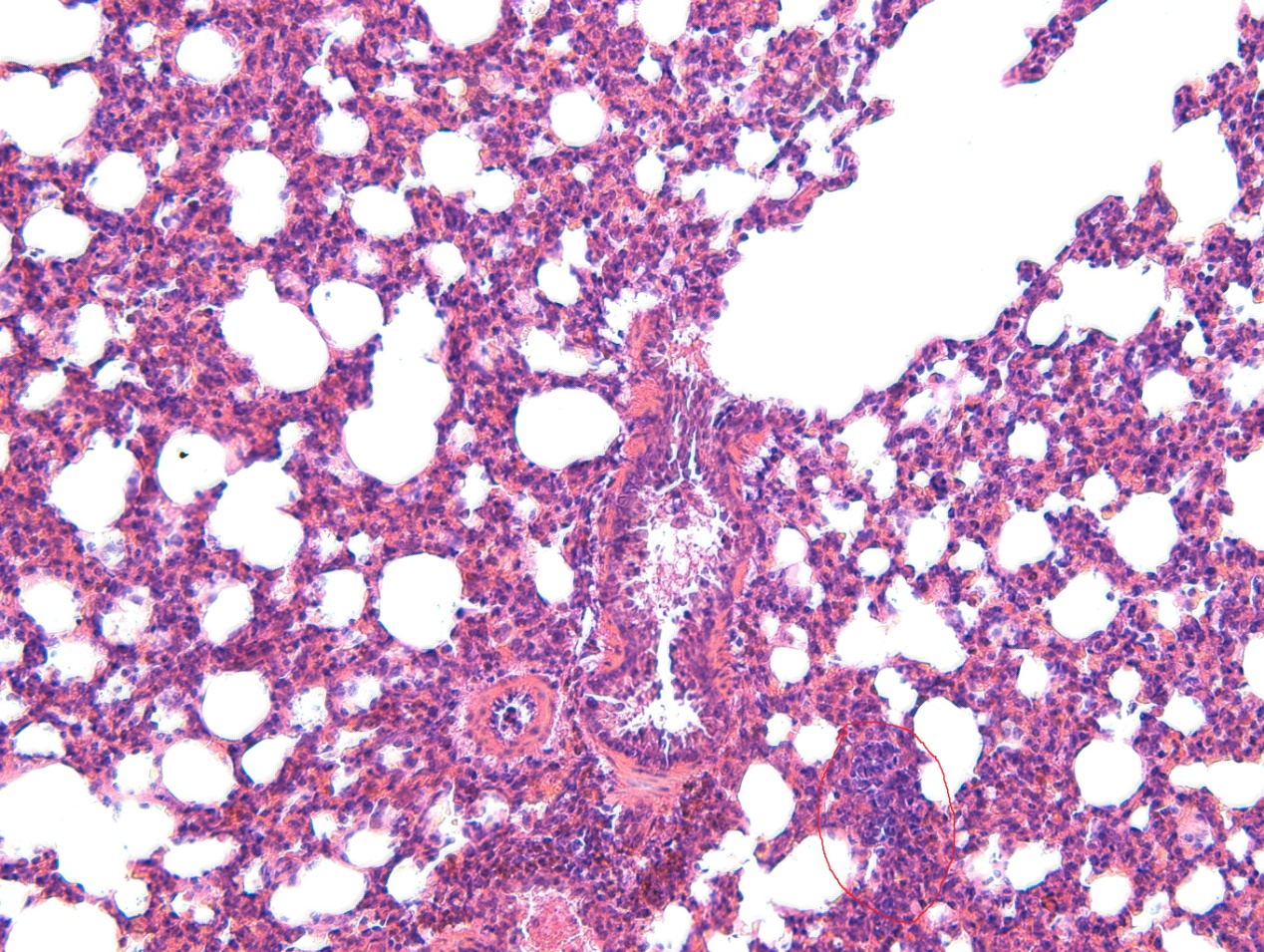

Supplement: Supplementary Materials — Supplementary Table S1: three-line table for cough symptoms of guinea pigs in six groups. Supplementary Table S2: three-line table for RI of guinea pigs in six groups. Supplementary Table S3: three-line table for Cydn of guinea pigs in six groups. Supplementary Table S4: three-line table for inflammatory cytokines of guinea pigs in six groups. Supplementary Table S5: three-line table for Ashcroft score of guinea pigs in six groups. Supplementary Table S6: three-line table for GR, total p38 MAPK, and p-p38 MAPK levels of guinea pigs in six groups. Supplementary Table S7: three-line table for mRNA MKP-1 level of guinea pigs in six groups. Supplementary Figure S1: levels of GR proteins in lung tissues. Supplementary Figure S2: levels of total p38 MAPK proteins in lung tissues. Supplementary Figure S3: levels of p-p38 MAPK proteins in lung tissues. Supplementary Figure S4: aggregation of inflammatory cells (red circle) in H&E staining slices from lung sections: (a) OVA group; (b) OVA + CS group. [file 1699429.f1.docx]
